# Prevalence of COVID‐19 vaccine reactogenicity among Bangladeshi physicians

**DOI:** 10.1096/fba.2021-00158

**Published:** 2022-03-24

**Authors:** Md. Anwarul Azim Majumder, Afzalunnessa Binte Lutfor, Ahbab Mohammad Fazle Rabbi, A. B. M. Muksudul Alam, Mizanur Rahman, Narayan Saha, Michael H. Campbell, Mainul Haque, Kamrun Nessa, Mohib Ullah Khondoker, Tapas Ranjan Das, Sayeeda Rahman, Fauzia Jahan, Saidur Rahman Mashreky, Abrar Wahab, Md. Tosaddeque Hossain Siddiqui, Karisha Hinkson‐Lacorbiniere, Roksana Ivy, Rezaul Islam, Yusuf Haider, Eliza Omar, S. M. Moslehuddin Ahmed, A. M. Selim Reza, A. K. M. Daud, Muiz Uddin Ahmed Choudhury, Md. Abed Hossain, Abdul Matin Pappu, Nusrat Jahan, Mohammed S. Razzaque

**Affiliations:** ^1^ Public Health Foundation of Bangladesh Dhaka Bangladesh; ^2^ The University of the West Indies Bridgetown Barbados; ^3^ Ad‐Din Women's Medical College Dhaka Bangladesh; ^4^ Bangladesh University of Textiles Dhaka Bangladesh; ^5^ Shaheed Suhrawardy Medical College Dhaka Bangladesh; ^6^ International Medical College Dhaka Bangladesh; ^7^ National Institute of Neurosciences & Hospital Dhaka Bangladesh; ^8^ National Defence University of Malaysia Kuala Lumpur Malaysia; ^9^ Chittagong Medical College Chittagong Bangladesh; ^10^ Gonoshasthaya Samaj Vittik Medical College Dhaka Bangladesh; ^11^ Sheikh Sayera Khatun Medical College Gopalganj Bangladesh; ^12^ American University of Integrative Sciences Bridgetown Barbados; ^13^ Bangladesh Medical College Dhaka Bangladesh; ^14^ Centre for Injury Prevention and Research Bangladesh (CIPRB) Dhaka Bangladesh; ^15^ Bangabandhu Sheikh Mujib Medical University Dhaka Bangladesh; ^16^ Shaheed Monsur Ali Medical College Hospital Dhaka Bangladesh; ^17^ Colonel Malek Medical College Manikganj Bangladesh; ^18^ Uttara Adhunik Medical College Dhaka Dhaka Bangladesh; ^19^ Jalalabad Ragib‐Rabeya Medical College Sylhet Bangladesh; ^20^ United Hospital Ltd Dhaka Bangladesh; ^21^ Lake Erie College of Osteopathic Medicine Pennsylvania USA

**Keywords:** AstraZeneca vaccine, Bangladesh, COVID‐19, physicians, reactogenicity

## Abstract

Increased COVID‐19 vaccine hesitancy presents a major hurdle in global efforts to contain the COVID‐19 pandemic. This study was designed to estimate the prevalence of adverse events after the first dose of the Covishield (AstraZeneca) vaccine among physicians in Bangladesh. A cross‐sectional study was conducted using an online questionnaire for physicians (*n* = 916) in Bangladesh. Physicians who received at least one dose of the COVID‐19 vaccine were included. The study was carried out from April 12 to May 31, 2021. More than 58% of respondents (*n* = 533) reported one or more adverse events. Soreness of the injected arm (71.9%), tiredness (56.1%), fever (54.4%), soreness of muscles (48.4%), headache (41.5%) and sleeping more than usual (26.8%) were the most commonly reported adverse events. Most vaccine‐related reactogenicities were reported by the younger cohorts (<45 years). The majority of respondents reported severity of reactogenicity as “mild,” experienced on the day of vaccination, and lasting for 1–3 days. The most common reactogenicity was pain at the injection site; the second most common was tiredness. Almost half (49.2%) of the physicians took acetaminophen (paracetamol) to minimize the effects of vaccine reactogenicity. Multivariate logistic regression analyses showed that physicians with diabetes and hypertension (OR = 2.729 95% CI: 1.282–5.089) and asthma with other comorbidities (OR = 1.885 95% CI: 1.001–3.551) had a significantly higher risk of vaccine‐related reactogenicities than physicians without comorbidities. Further safety studies with larger cohorts are required to monitor vaccine safety and provide assurance to potential vaccine recipients.

## 1 INTRODUCTION

Recent studies have demonstrated that high rates of COVID‐19 vaccine hesitancy among both the general population and healthcare professionals (HCPs) present a major hurdle in global efforts to contain the COVID‐19 pandemic.^1^, ^2^, ^3^, ^4^, ^5^ Public dissemination of evidence for the safety and efficacy of vaccines may encourage vaccine acceptance.^2^ In the absence of sufficient vaccine acceptance, universal access to vaccination may not achieve immunization coverage essential to control the ongoing pandemic.^6^ In fact, global herd immunity (population vaccine coverage of 60%–80%) is becoming unachievable due to stark disparities in vaccination rates among different countries.^7^, ^8^ As of 23 February 2022, more than 4.9 billion vaccine doses have been administered worldwide, which is equal to 63.9% of the world population.^7^

Several countries have temporarily discontinued the Oxford‐AstraZeneca vaccine over concerns that the vaccine may be linked to an increased risk of blood clots.^9^ Although blood clots have been reported as an infrequent side effect in some populations, the risk of clotting due to COVID‐19 infection appears to be greater than that posed by the vaccine. Nonetheless, these concerns may contribute to vaccine hesitancy.^10^, ^11^, ^12^ In addition to these rare, serious complications, more commonly reported symptoms associated with reactogenicity may also contribute to vaccine reluctance.

The reactogenicity of COVID‐19 vaccines is emerging.^13^ Thus far, data on vaccine safety and adverse events has been obtained primarily from manufacturer‐sponsored studies.^14^ A few clinical trials have published short‐term findings of the efficacy and safety of COVID‐19 vaccines.^13^ Various government agencies monitor vaccine reactogenicity to rapidly detect safety ranges and rare adverse events, as well as provide real‐time data for risk analysis and decision‐making.^15^, ^16^, ^17^ For example, the U.S. Centers for Disease Control (CDC)^16^, ^17^ and the U.K. Medicines & Healthcare products Regulatory Agency (MHRA)^18^ collect self‐reported data from vaccine recipients via online tools.^19^ Despite the recognized challenges of self‐reporting symptoms, including inconsistency of data, reporting biases and lack of control groups,^20^ health authorities frequently use this approach to make inferences about the wider population of vaccine recipients.^16^, ^17^, ^18^ Bangladesh started vaccination for COVID‐19 from 8 February 2021. To our knowledge, this study is the first to report the prevalence and severity of COVID‐19‐vaccine associated reactogenicity among physicians in Bangladesh.

The current study aimed to estimate the prevalence of the AstraZeneca vaccine reactogenicity among physicians who received vaccinations in the initial phase of vaccine roll‐out in Bangladesh. We surveyed only physicians and excluded other contemporary vaccine recipients to document reactogenicity in professionals with training to identify and clearly articulate symptoms. Monitoring the reactogenicity of COVID‐19 vaccines has the potential to identify uncommon adverse responses particular to Bangladeshi cohorts. Documenting reactogenicity is crucial for planning necessary clinical supports following COVID‐19 vaccination in Bangladesh and establishing safety data to promote vaccine acceptance.

## 2 MATERIALS AND METHODS

### 2.1 Study design and participants

A cross‐sectional survey was conducted among physicians working in different government and private sector academic institutes and hospitals in Bangladesh. Inclusion criteria were physicians who received at least one dose of the AstraZenica COVID‐19 vaccine. The study was conducted from 12 April 2021 to 31 May 2021.

### 2.2 Data collection

We asked physicians to complete a self‐administered online survey (via the Google Docs^®^ platform) adapted for Bangladesh from an instrument developed by researchers working in Barbados (Hinkson‐Lacorbiniere and team). The questionnaire was validated by a multinational panel of public health specialists and amended as per their suggestions. A pilot study was conducted among 29 respondents who were excluded from the formal evaluation, and further adjustment was done based on their inputs.

The modified questionnaire included demographic information, vaccination status (single dose or both required doses), history of COVID‐19 infection and presence of comorbidities (including diabetes, hypertension, lung disease, kidney disease and cancer). Vaccine reactogenicity was recorded in terms of time of symptom onset (same day, 1–3 days post‐vaccination, 4–7 post‐vaccination), severity (*Severe*—I had to seek medical attention; Moderate—I had to stop my daily activities; *Mild*—I was still able to do most daily activities), duration (1 day, 2–3 days, 4–7 days, still present) and whether treatment measures were taken (yes, no). Additionally, the questionnaire elicited physicians’ awareness of thromboembolic events and thrombocytopenia following vaccination.

The survey was conducted and reported based on the checklist for reporting results of internet e‐surveys (CHERRIES).^21^ Because the survey was time sensitive, we recruited participants using convenience sampling by sharing the survey link via social networks (Facebook, Messenger, WhatsApp and Viber) and e‐mail. Investigators took the advantage of social media groups, professional associations and healthcare organizations to promote the survey.

Participation in the survey was voluntary and anonymous. All the participants gave consent before participation. No identifiable personal information was collected or stored.

### 2.3 Ethical approval

Prior ethical approval was granted by the Research Ethics Committee of Shaheed Suhrawardy Medical College, Dhaka, Bangladesh (No: ShSMCH/Ethical/2021/09).

### 2.4 Statistical analysis

We calculated the reported prevalence of reactogenic events and their relationship with recorded demographic information. The primary outcome variable of interest was the presence of reactogenicity following COVID‐19 vaccination. Further, bivariate analyses were performed to examine the link between existing comorbidities, demographic characteristics and reported adverse events. Multivariate logistic regression was performed to investigate the individual effects of predictor variables on reactogenic symptoms. All statistical analysis was performed using IBM SPSS 22.

## 3 RESULTS

### 3.1 Responders’ characteristics

The demographic characteristics of the participants are shown in Table 1. A total of 916 physicians completed the questionnaire. The majority of respondents were male (52.8%) and were employed in the public/government sector (60.6%). Many of the respondents (35%) were those between 31‐40 years. More than half of the respondents (52.2%) reported no history of chronic diseases. More than a quarter of respondents (28.5%) had tested positive for COVID‐19 infection, and about three‐quarters (78.3%) had received both first and second doses of COVID‐19 vaccination at the time of the survey. All participants received the Covishield (AstraZeneca) vaccine, which was the only available vaccine in Bangladesh during the study period.

**TABLE 1:** Demographic and background information of study respondents (*n* = 916)

Variables	Number of observations	Percentages
Gender of respondent		
Male	484	52.8
Female	432	47.2
Age of respondents (in years)		
21–30	142	15.5
31–40	321	35.0
41–50	233	25.4
51–60	161	17.6
61–70	52	5.7
71–80	1	0.1
Workplace of respondent		
Private	344	37.6
Public/government	555	60.6
Other research institutions	14	1.5
Work type of respondents (detailed)		
Medical colleges/universities and affiliated hospitals	491	53.6
Government Hospitals	210	22.9
Private hospitals	119	13.0
Others	96	10.5
Vaccination status		
First dose only	193	21.1
Both first and second doses	717	78.3
COVID−19 test status		
Tested positive (RT‐PCR)	261	28.5
Never tested	58	6.3
No	591	64.5
Timing of getting infected with COVID−19		
Before the 1st dose	200	21.8
Between 1st dose and 2nd dose	68	7.4
After the 2nd dose	5	0.5
Prior presence of any chronic illness^a^		
No illness	478	52.2
Diabetes	31	3.4
Diabetes; Hypertension	45	4.9
Diabetes; Hypertension and other comorbidities	24	2.6
Diabetes and other comorbidities	15	1.6
Hypertension and other comorbidities	164	17.9
Obesity and other comorbidities	39	4.3
Asthma and other comorbidities	63	6.9
Other comorbidities	32	3.5
Measures take to alleviate adverse effects^a^		
Drug taken: Paracetamol	451	49.2
Drug taken: Ibuprofen	10	1.1
Drug taken: Other pain killer	20	2.2
Cold bath/shower/sponge	51	5.6
Sleep	212	23.3
Drinking more water	205	22.4
Nothing worked	24	2.6
Nothing taken	42	4.6
Other actions	13	1.4
Experienced similar adverse effects from other vaccines (e.g. BCG, HPV)		
Yes	104	11.4
No	390	42.5
Don't remember	422	46.1
Awareness: Risk of blood clotting after vaccination		
Yes	690	75.3
No	145	15.8
Don't know	81	8.8
Awareness: Risk of low platelets (thrombocytopenia) after vaccination		
Yes	506	55.2
No	278	30.3
Don't know	132	14.4

^a^ Multiple answers.

### 3.2 Prevalence of vaccine reactogenicity

**FIGURE 1:**
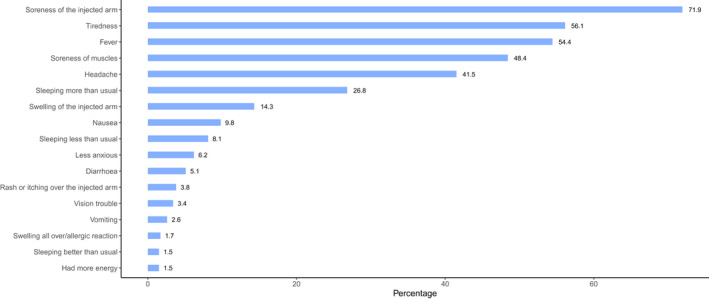
Prevalence of reactogenicity among respondents after receiving the first dose of Covishield (AstraZeneca) vaccine

The prevalence of vaccine reactogenicity among respondents is shown in Figure 1. More than 58% (*n* = 533) respondents reported one or more reactogenic symptoms. The six most commonly reported adverse events were “soreness of the injected arm” (71.9%), “tiredness” (56.1%), “fever” (54.4%), soreness of muscles” (48.4%), “headache” (41.5%) and “sleeping more than usual” (26.8%). Most respondents characterized the severity of symptoms as mild. However, some respondents did rate their experience of symptoms as severe. The most common severe symptoms were fever (9.4%) and tiredness (20.1%). Only 11.5% of the respondents recalled similar adverse events from previous vaccinations for other diseases (e.g., BCG, HPV). Approximately half (49.2%) of the respondents took acetaminophen to treat reactogenic symptoms. Other actions taken to treat symptoms were sleep (23.1%) and drinking water (22.4%). More than 75% of the respondents were aware of the risk of thromboembolic events, and more than half (55.5%) were mindful of thrombocytopenia.

The observed types of reactogenicity, including onset and duration, are summarized in Table 2. For most respondents, these adverse events appeared on the same day of vaccination, except for tiredness (24%), which appeared 2–3 days post vaccination. For 46.5% of participants, soreness in the arm occurred on the same day of vaccination; same‐day fever was reported by 34.3% of respondents. However, most respondents reported duration of 1–3 days for these frequently observed reactogenicities. For 45.8% of participants, soreness in the arm lasted for 1–3 days, followed by fever (31.5%). Tiredness persisted for 7 days for 7.7% of participants and beyond 7 days for 3.9%.

**TABLE 2:** Summary of six most commonly reported reactogenic symptoms (*n* = 533)

Adverse effect	The severity of adverse events				Time of appearance				Duration adverse events last				
	Severe	Moderate	Mild	Total	That same day	Between 2–3 days	Between 4–7 days	Total	Same day	1–3 days	4‐7days	>7 days	Total
Soreness of the injected arm	30 (5.6%)	74 (13.9%)	279 (52.3%)	383 (71.9%)	248 (46.5%)	128 (24.0%)	6 (1.1%)	382 (71.7%)	57 (10.7%)	244 (45.8%)	65 (12.2%)	6 (1.1%)	366 (68.7%)
Soreness of muscles	27 (5.1%)	84 (15.8%)	147 (27.6%)	258 (48.4%)	132 (24.8%)	106 (19.9%)	3 (0.6%)	241 (45.2%)	39 (7.3%)	160 (30.0%)	30 (5.6%)	6 (1.1%)	229 (43.0%)
Fever	50 (9.4%)	87 (16.3%)	153 (28.7%)	290 (54.4%)	183 (34.3%)	96 (18.0%)	2 (0.4%)	281 (52.7%)	87 (16.3%)	168 (31.5%)	15 (2.8%)	6 (1.1%)	276 (51.8%)
Headache	34 (6.4%)	63 (11.8%)	124 (23.3%)	221 (41.5%)	125 (23.5%)	74 (13.9%)	4 (0.8%)	203 (38.1%)	49 (9.2%)	124 (23.3%)	23 (4.3%)	15 (2.8%)	211 (39.6%)
Tiredness	35 (6.6%)	107 (20.1%)	157 (29.5%)	299 (56.1%)	107 (20.1%)	128 (24.0%)	11 (2.1%)	246 (46.2%)	42 (7.9%)	127 (23.8%)	41 (7.7%)	21 (3.9%)	231 (43.3%)
Sleeping more than usual	16 (3.0%)	57 (10.7%)	70 (13.1%)	143 26.8%)	64 (12.0%)	54 (10.1%)	4 (0.8%)	122 (22.9%)	36 (6.8%)	56 (10.5%)	18 (3.4%)	19 (3.6%)	129 (24.2%)

The prevalence of reactogenicity among physicians stratified by gender and age is shown in Table 3. Females reported a higher incidence of reactogenicity compared to males. Fever, vision trouble, sleeping more than usual, rash/itching over the injected arm, and nausea were significantly more common among females (*p* < 0.05). Most of the adverse events were reported by respondents <45 years, irrespective of gender. Adverse events classified as “other” are shown in Appendix 1. Four case studies describing these reports are contained in Appendix 2.

**TABLE 3:** Prevalence of reactogenic symptoms among physicians stratified by gender and age

Adverse events	Gender				Age			
	Male (*n* = 269)	Female (*n* = 264)	Total	*p*‐value	21–44 years old^a^ (*n* = 370)	45+ years old^b^ (*n* = 160)	Total	*p*‐value
Soreness of the injected arm	200 (74.3%)	199 (75.4%)	399	0.518	303 (81.9%)	94 (58.8%)	397	0.092
Soreness of muscles	136 (50.1%)	127 (48.1%)	263	0.678	197 (53.2%)	64 (40.0%)	261	0.486
Fever	147 (54.7%)	147 (55.7%)	294	0.007*	218 (58.9%)	74 (46.3%)	292	0.101
Headache	114 (42.4%)	110 (41.7%)	224	0.257	168 (45.4%)	56 (35.0%)	224	0.398
Vision trouble	6 (2.2%)	13 (4.9%)	19	0.039*	16 (4.3%)	3 (1.9%)	19	0.729
Tiredness	148 (55.0%)	159 (60.2%)	307	0.090	236 (63.8%)	70 (43.8%)	306	0.919
Sleeping more than usual	66 (24.5%)	83 (31.4%)	149	0.076	118 (31.9%)	30 (18.8%)	148	0.407
Sleeping less than usual	22 (8.2%)	22 (8.3%)	44	0.925	31 (8.4%)	13 (8.1%)	44	0.039*
Sleeping more than usual	3 (1.1%)	8 (3.0%)	11	0.038*	7 (1.9%)	4 (2.5%)	11	0.750
Had more energy	6 (2.2%)	4 (1.5%)	10	0.422	9 (2.4%)	1 (0.6%)	10	0.558
Less anxious	21 (7.8%)	14 (5.3%)	35	0.103	25 (6.8%)	10 (6.3%)	35	0.241
Swelling of the injected arm	31 (11.5%)	48 (18.2%)	79	0.023	69 (18.6%)	10 (6.3%)	79	0.164
Swelling all over/allergic reaction	3 (1.1%)	6 (2.3%)	9	0.407	7 (1.9%)	2 (1.3%)	9	0.912
Rash/itching over the injected arm	4 (1.4%)	16 (6.0%)	20	0.003*	15 (4.0%)	5 (3.2%)	20	0.768
Diarrhea	12 (4.4%)	16 (6.0%)	28	0.499	19 (51.4%)	8 (5.0%)	27	0.464
Nausea	20 (7.43%)	33 (12.5%)	53	0.038*	44 (11.9%)	9 (5.6%)	53	0.586
Vomiting	6 (2.2%)	9 (3.4%)	15	0.426	13 (3.5%)	2 (1.3%)	15	0.915

^a^ 21‐44 years: Younger participants.

^b^ 45+ years: Older participants.

* Significance: *p* < 0.05.

### 3.3 Determinants of adverse events

Findings from binary logistic regression analyses are presented in Table 4. All age groups had a significant impact on having adverse events than the physicians with younger age group. Physicians aged 61–70 years were almost 96% less likely to have an adverse event than physicians in their twenties (OR = 0.041 with 95% CI lies between 0.016 and 0.105). Existing comorbidity has an impact on having adverse events as well. Physicians with diabetes and hypertension were 2.72 times more likely to have an adverse event than physicians without prior conditions. Asthma and other comorbidities (OR = 1.885 95% CI: 1.001–3.551) also significantly increased the risk of reactogenicities than physicians without comorbidities.

**TABLE 4:** Logistic regression coefficients and odds ratios (95% CI) for determinants reactogenic symptoms

Variables	β	SE (β)	Exp(β) with 95% CI
Gender of respondent			
Male (ref)			
Female	−0.007	0.158	0.993 (0.729, 1.353)
Age of respondents (in years)			
21–30 (ref)			
31–40	−0.762**	0.264	0.467 (0.278, 0.783)
41–50	−1.243***	0.280	0.289 (0.167, 0.500)
51–60	−1.842***	0.321	0.159 (0.084, 0.298)
61–70	−3.205***	0.484	0.041 (0.016, 0.105)
Work type of respondents (detailed)			
Medical college/hospital (ref)			
Medical university/hospital	−0.077	0.294	0.926 (0.521, 1.647)
Private hospital	0.223	0.245	1.250 (0.773, 2.021)
District hospital	0.802*	0.418	2.231 (0.984, 5.058)
Government specialized hospital	−0.306	0.249	0.737 (0.452, 1.200)
Upazilla health complex	0.571	0.372	1.771 (0.855, 3.669)
Institute of health technology	0.924	1.121	2.520 (0.280, 22.677)
Dental college	−1.492	1.218	0.225 (0.021, 2.446)
Others	−0.039	0.276	0.962 (0.560, 1.651)
Prior presence of any chronic illness			
No illness (ref)			
Diabetes	0.130	0.434	1.139 (0.486, 2.667)
Diabetes; Hypertension	1.004**	0.385	2.729 (1.282, 5.089)
Diabetes; Hypertension and other diseases	0.304	0.457	1.356 (0.554, 3.319)
Diabetes and other diseases	0.726	0.614	2.066 (0.620, 6.880)
Hypertension and other diseases	0.194	0.213	1.214 (0.799, 1.842)
Obesity and other diseases	0.707*	0.422	2.027 (0.886, 4.636)
Asthma and other diseases	0.634*	0.323	1.885 (1.001, 3.551)
Other diseases	0.483	0.435	1.621 (0.691, 3.802)

Note

Reference category is denoted by (ref). Significance: ***p < 0.01, **p < 0.05, *p < 0.1.

## 4 DISCUSSION

The study estimated the prevalence of reactogenicity after the first dose of the AstraZeneca vaccine among Bangladeshi physicians. To the best of our knowledge, this is the first study of its type in Bangladesh. A key strength of this survey is the accuracy and reliability of symptom reporting by medical professionals.^22^, ^23^ We found that over half (58.2%) of respondents reported at least one reactogenic side effect after the first dose of vaccine. Two studies of the general population in Bangladesh at approximately the same time as the current study reported similar prevalence of adverse events: 50.9% in February‐June 2021^24^ and 54.1% in May 2021.^25^ Compared to Bangladesh, higher vaccine reactogenicity has been reported in studies of HCPs in India (65.9%^26^ and 69.7%^27^), South Korea (99.8%,^28^ 98.1%,^29^ 90.9%,^30^ and 93%^31^), Germany, Czech Republic (94.6%),^32^ Togo (71.6%),^33^ Nepal (85%),^34^ Saudi Arabia (96.1%),^35^ Ethiopia (68.4%)^36^ and Ghana (80.7%).^37^ However, lower rates were found among HCPs in two studies from India—40%^38^ and 56.9%.^22^ These disparities may be due to greater representation of elderly participants (≥65 years), as older adults generally exhibit milder symptoms.^29^ Jeon et al.^29^ noted the higher incidence (0% vs. 8.9%) and greater severity of reactogenic events in a younger age group compared to a study conducted by Voysey et al.^39^ with participants ≥65 years. In the present study, 5.8% of respondents were ≥60 years old, which may be one of the reasons for lower reported adverse events. Further, our study found that physicians aged 61–70 years were almost 96% less likely to have adverse events than physicians in their twenties. Similar age‐related findings were reported in other studies of Covisheild,^32^, ^36^ Pfizer‐BioNTech and Moderna vaccine recipients.^40^

Reactogenicity is usually induced by innate and adaptive immune responses leading to the release of chemokine and cytokines. Reactogenic symptoms are the result of chemokines and cytokines that mimic systemic immune response and include fever, tiredness, fatigue, pain and headache. Similarly, the release of inflammatory mediators due to immune response at the injection site leads to local reactions. These symptoms are evidence of effective vaccination.^41^ The most commonly reported reactogenicity in our study was pain at the injection site, which was more prevalent among females and younger respondents. These findings are consistent with those of previous studies on the vaccination of HCPs. The most common reactogenicity reported in our study coincides with other studies conducted among HCPs^26^, ^29^, ^33^, ^34^, ^42^ and studies conducted among the general population.^19^, ^43^ As in other studies,^26^, ^27^, ^29^, ^33^, ^34^, ^42^, ^44^ most of our respondents experienced mild symptoms that were self‐limiting and resolved within a few days (1–3 days). Approximately half of the respondents took acetaminophen to treat symptoms, which is more than reported in other recent studies. One‐quarter of respondents in a general‐population Bangladeshi study used acetaminophen to minimize vaccine‐associated discomfort,^25^ as did 33.3% of HCPs in an Ethiopian study.^36^

We found that fever, vision trouble, sleeping more than usual, rash/itching over the injected arm and nausea were more commonly reported by females (*p *< 0.05). From the detailed frequency distribution, we found that female physicians experienced vaccine reactogenicity earlier than their male counterparts and that symptoms usually disappeared within 1–3 days in female physicians (not shown in tables of result section). Studies demonstrated that increased experience of adverse vaccination‐related events in women is related to estradiol, which can induce a more robust immune responses following vaccination.^45^, ^46^ Females typically exhibit higher innate, humoral and cellular immune responses to viral infections as well as in response to vaccines.^47^ Specific manifestations of gender differences in immune response have been documented in several studies. Females tend to have more robust immune responses due to greater generation of antibodies and a more robust T‐cell response.^48^ Further, females exhibit higher levels of antibody response, humoral response and cell‐mediated immune response to antigenic stimulation, vaccination and infection.^49^ This higher vaccine reactogenicity is associated with higher basal and post‐vaccination IgG levels and increased B cell numbers and functions compared to men.^47^, ^50^ Finally, higher body fat content in females may reduce the distribution and clearance of medications.^51^

We found that existing comorbidities increased the likelihood of adverse reactogenic events. Physicians with “diabetes and hypertension” and “obesity and other complications” had a double risk of reactogenicity. A recent general‐population Bangladeshi study reported similar findings with an odds ratio of reactogenic symptoms after the first vaccine dose of 1.8 for participants with comorbidities. An Ethiopian study of HCPs also found that the presence of comorbidities doubled the risk of reactogenicity.^36^ Despite an increased risk of adverse vaccine reactions, people with underlying medical conditions are also at increased risk of COVID‐19 infections.^52^ The World Health Organization (WHO) Strategic Advisory Group of Experts (SAGE) on Immunization clinical trials with the Oxford‐AstraZeneca (Covishield) vaccine (AZD1222) concluded that people with comorbidities (obesity, cardiovascular disease, respiratory disease and diabetes) had an increased risk of severe COVID‐19.^53^ For most people with comorbidities, the benefits of COVID‐19 vaccination outweigh the risks of adverse events.

In Appendix 1, we list the adverse events reported by respondents in the ‘other’ categories. Earlier studies have not revealed some of these infrequent adverse events (e.g., cracked teeth, meningismus, severe eye pain, menstrual irregularities including spotting, excessive menstrual bleeding, decreased urine output and hematuria). Because these are idiosyncratic events, their clinical significance is unclear. We present four case studies of clinically significant adverse events (Appendix 2). One of the surveyed physicians complained of sudden vertigo and lost consciousness for a few seconds which occurred 2.5 h following vaccination. After regaining consciousness, ECG suggested acute myocardial infraction, and the physician required surgical intervention for blockage in the left anterior descending artery. Another physician reported menstrual irregularities with spotting lasting for 15 days. Severe neck pain and severe pain while walking (spasm of bilateral quadriceps muscles) were experienced by another physician. The last case study describes the experience of vertigo and orthostatic hypotension starting immediately after vaccination and persisting for 3 days. These unusual complications should be carefully documented, but their relationship to immunization is not established. Similarly, a UK‐based phase 2/3 trial identified 13 serious adverse events (SAEs), but none were established to be related to vaccination.^54^ Voysey et al.^39^ reported 175 SAEs occurring in 168 of 11,636 participants, of which only three events were shown to be related to vaccination.

### 4.1 Study limitations

Because of sampling limitations, there is a possibility that survey results might not generalize to the entire HCP population of Bangladesh. However, since all participants were physicians, we believe that their reporting of reactogenicity is exceptionally accurate. This study design explicitly does not address the general population. Broader multicentric studies are required to obtain a true picture of reactogenicity in the general population after both or booster doses of vaccination. Additionally, we evaluated only short‐term reactogenicity, and surveillance will be needed to determine possible long‐term effects of vaccination. More robust probability sampling will provide better understanding of prevalence and underlying causes of reactogenic and other adverse vaccination‐related events.

## 5 CONCLUSION

The majority of vaccine recipients in our study reported reactogenicity, but symptoms were mild and of short duration. The most common reactogenic symptoms were pain at the injection site and tiredness. Reactogenicity was reported more frequently among females and younger age groups. Vaccine recipients and healthcare staff should be aware of possible reactogenicity and management protocols to ensure that vaccination benefits are maximized relative to risks. Further studies on vaccine safety are required for monitoring and to assure the public regarding safety of available vaccines.

## CONFLICT OF INTEREST

The authors have no conflict of interest to declare.

## AUTHOR CONTRIBUTIONS

Majumder MAA, Lutfor AB, Razzaque MS, Alam ABMM: planned & designed the study. Lutfor AB, Alam ABMM, Majumder MAA, Siddiqui MTH, Nessa K, Khondoker MU, Rahman M, Saha N, Jahan F, Ivy R, Islam R, Haider Y, Haque M, Omar E, Ahmed SMM, Reza AMS, Daud AKM, Choudhury MUA, Hossain MA, Rahman S, Pappu AM: actively collected the data. Majumder MAA, Lutfor AB, Razzaque MS, Mashreky SR, Rahman S, Rabbi AMF, Wahab A: wrote the manuscript. Rabbi AMF, Mashreky SR, Wahab A, Majumder MAA: analyzed the data. Hinkson‐Lacorbiniere K: developed the original questionnaire and edited the manuscript. Majumder MAA, Lutfor AB, Razzaque MS, Mashreky SR, Rahman S, Rabbi AMF & Wahab A: Modified the questionnaire. Campbell MH: critically read the manuscript, edited the manuscript & provided useful suggestions on data analysis. All authors: critically reviewed the manuscript and approved the final draft. Majumder MAA, Lutfor AB, MuAlam ABMM have full access to all the data and take responsibility for the integrity of the data.

## APPENDIX 1 Adverse events reported in the “other” category

**Table:**

Vertigo (*n* = 3) Orthostatic hypotension (*n* = 1) Myalgia (*n* = 2) Severe backache (*n* = 2) Cracked teeth (*n* = 1) Muscle cramp (*n* = 2) joint pain (*n* = 2) Sore throat (*n* = 2) Meningismus (*n* = 1) Severe eye pain (*n* = 1) Severe back ache radiating to both legs (*n* = 1)	Dizziness (*n* = 1) Abdominal pain (*n* = 1) Menstrual irregularities including spotting (*n* = 1) Excessive menstrual bleeding (*n* = 1) Decreased urine output (*n* = 1) Migraine (*n* = 1) Palpitation (*n* = 2) Hematuria (*n* = 1) Severe thirst (*n* = 1) Unconsciousness (*n* = 1)

## APPENDIX 2 Four case studies related to severe adverse events

**Table:**

**Case study 1** Age: 63 years Gender: Male Co‐morbidity: Diabetes Vaccine status: First dose only The participant received the vaccine at about 11 a.m. on 13.02.2021. About 2.5 h later, he had sudden vertigo with the tingling whole of the left upper extremity and immediately became unconscious for a few seconds. After gaining consciousness, he had severe bouts of vomiting. The participant had no chest pain, no compression chest, no dyspnoea, but exhibited profuse sweating. Tingling left upper extremity was persisting. He had an ECG with very high ST elevations suggesting acute myocardial infarction (AMI). He took Ecosprin 4, clopidogrel 4, Emistat 1 & anti‐ulcerent. He then was rushed to Dhaka (capital city). On his way, initially, he felt chest compression and took Nitroglycerin sublingually. When he arrived in Dhaka, all of his discomforts disappeared, and he started to feel quite better. He had an angiogram which revealed two long segment blocks in the left anterior descending artery. Two days later, two stentings were done, and the patient returned home 2 days after surgery. *Past history*: He never had any dyspnoea, chest pain, compression, any sudden sweating or discomfort on going up (4 to 7/8 stairs at stress). He was a chain smoker of 20+ sticks/day. He was taking no medicine to control diabetes, which was always 10–14 mmol/L since 2006. He was not on exercise for diabetes mellitus.
**Case study 2** Age: 28 years Gender: Female Co‐morbidity: No illness Vaccine status: First dose only Her menstrual cycle changed. The menstrual cycle started 15 days after vaccination and lasted for 15 days. She had not experienced this previously. She was also experiencing spotting.
**Case study 3** Age: 30 years Gender: Male Co‐morbidity: Nonalcoholic fatty liver disease Vaccine status: Taken both dosages The participant experienced severe neck pain and had severe pain and spasms of the bilateral quadriceps muscles, causing unbearable pain while walking. The pain was relieved by hot compression, analgesics and topical Vollygel (diclofenac gel) application on affected muscles.
**Case study 4** Age: 34 years Gender: Male Co‐morbidity: No illness Vaccine status: Taken both dosages The respondent experienced vertigo and orthostatic hypotension, which started immediately after vaccination and lasted for 3 days.
